# Clinical Profiles of Dengue Fever Patients, during an Outbreak

**Published:** 2019-06-24

**Authors:** Shafia Saba, Ata Ur Rehman Khan, Unsar Naeem-Ullah, Syed Haroon Masood Bokhari

**Affiliations:** 1 Department of Entomology, Muhammad Nawaz Shareef University of Agriculture, Multan, Pakistan; 2 Epidemic Prevention and Control Program, District Health Authority, Punjab, Pakistan

**Keywords:** Dengue fever, Outbreak, Public health, Vector-borne diseases

## Abstract

**Background:**

Dengue fever (DF) has become a major public health concern globally. It is an infection caused by a virus of the family Flaviviridae, with five serotypes (DENV 1–5). Recent years have seen an increase in the prevalence of the disease in Pakistan. The current study was carried out to evaluate the clinical features, laboratory findings and demographic information of the patients reported during the dengue outbreak in Multan of Pakistan in 2015.

**Methods:**

The hospital documentation-based data of confirmed DF cases were collected for the 6 months period from a Tertiary Care Hospital in Multan, Pakistan. The patients were labeled as confirmed on the basis of NS1 and IgM positivity by ELISA. The data collected were analyzed using SPSS.

**Results:**

Overall, 361 patients were investigated (78.67% males and 21.33% females), with maximum infection rate in the age group of 18–35yr (50.41%). Mean hospital stay was 2.64d (SD 1.2), while mean fever duration was 5.27 (SD 1.57). Outbreak occurred during the months from Jul–Dec, while maximum patients were reported in Oct (287). No mortality was reported, and all patients recovered.

**Conclusion:**

Better management practices and timely reporting can reduce the risk factors associated with the disease.

## Introduction

Our planet is going through a critical phase due to vector-borne diseases (VBDs) of humans. Besides others, arthropods are the major group to transmit diseases in human. Among the VBDs spread by insects, dengue fever (DF) is the most prevailing disease in human communities after malaria, with vast geographic distribution (1). Primary vector responsible for transmission of the disease is mosquito species *Aedes aegypti*, followed by *Ae. albopictus* (2), and the rate of infection is higher in rainy months/days because of increasing populations and breeding sites of vector mosquitoes (3).

Dengue fever is a febrile disease also known as “break-bone fever” (4) caused by a virus of the family Flaviviridae (genus *Flavivirus*) (5) with five serotypes viz. DEN-1, DEN-2, DEN-3, DEN-4 and DEN-5 (6). DEN-1 and DEN-3 have been stated to cause more serious primary infections while remaining are extra problematic and serve as a source of secondary infection (7). Once, the patients have any of the said serotypes they cannot get infected with the same again, but the situation goes worst as the patient lose self-immunity against the other serotypes. However, the condition becomes more critical when individuals get second infection (after first infection), thus resulting in extreme illness (8).

The disease can occur in more than one forms ranging from dengue fever (DF), dengue hemorrhagic fever (DHF) and dengue shock syndrome (DSS) (9). The symptoms shown by patients of DF are fever, musculoskeletal pain, retrobulbar pain, headache, and morbilliform rashes. DHF patients have high-grade fever and hemorrhagic attacks. DSS is characterized by negative change in mental situation and normally low blood pressure (10). Classical dengue is reported mainly in children, youngsters, and adults (11).

The worldwide spread of the disease has increased radically in past few years with half of the world population under the peril of infection and it is claimed that 3.9 billion people inhabiting 128 countries are under the threat of Df (12). About 50–100 million cases are reported every year and the death rate in various countries is not less than twelve thousand per year (1). The disease has spread to more areas over time; for example, before 1970 the DHF was endemic only in nine countries. Whereas during the year 1995, the number increased more than four times and in 1998 about 2500 million people were at verge of the disease (5). High numbers of cases were from tropical countries with poor economies (13). Initially, dengue cases were only recorded from urban areas but in recent past cases have been reported from rural areas as well (14).

Travelers are responsible to disseminate the disease from across the countries. A large proportion of people who traveled through tropical and sub-tropical countries acquired the disease. This condition alone increased the dengue cases up to 16% by 2005. Now dengue is reported more than malaria in travelers from countries of South East Asia (15). Since last many years, Pakistan is facing regular epidemics in one or another area in all of its provinces during and soon after rainy seasons (16). The disease was reported in the country for the first time in 1985, in youth of 16yr with a strange fever (17). In Hub, Baluchistan in 1995, 75 dengue cases were reported out of which 57 doomed to death (18). In 2011, Pakistan faced the worst strike of dengue in which more than 300 deaths and 20000 cases were reported (19). Among the affected cities, Lahore was severely hit followed by Faisalabad, Multan, Bahawalpur and Sargodha (20). In 2015, the Multan District of Punjab witnessed a devastating outbreak of dengue fever in all towns of the district.

The objective of present study was to analyze vulnerability of local people for the disease in various demographic traits like gender and age, geographical prevalence of the patients in the district, monthly trend of infection and clinical findings of the patients reported in a tertiary care hospital.

## Materials and Methods

### Description of the study area

The historic city of saints “Multan” is situated on the bank of “Chenab River” and lies at 30°11′52″N latitude and 71°28′11″E longitude in Punjab Province of Pakistan. Multan is the 7^th^ most populous city of Pakistan and is the cultural and economic center of South Punjab. Its total area is 781km^2^ with highest elevation of 423ft. The District Multan comprises 6 autonomous towns including Shah Rukn-e-Alam (SRA), Musa Pak Shaheed (MPS), Sher Shah (SS), Bosan, Shujabad (SB) and Jalal Pur Pirwala (JPP) town. Population of the district consists of 1.871 million inhabitants. The climate of the district is hot and dry. The summer season starts from May and continues till September. May, June, and July are the hottest months. Rainfall occurs during monsoon i.e., from July to Sep, while July is the wettest month. During winter season there is very little rain (21).

The current study was carried out with the collaboration of Epidemic Prevention and Control Program, District Health Authority, Multan by collecting data of patients reported in Nishtar Hospital Multan from Jul to Dec 2015.

### Collection of Clinical Data

A hospital documentation-based study was conducted from 1^st^ July 2015 to 31^st^ Dec 2015 in Nishtar Hospital Multan, Punjab, Pakistan. The data regarding patients reported with fever and other symptoms related to dengue from all above-mentioned towns of Multan was included in the study. The DEAG (Dengue Expert Advisory Group) case definitions were used as a guideline to label the patients as suspect, probable and confirmed Dengue Fever Patients (22). However, only the confirmed patients were enrolled. The patients were labeled as Confirmed DF on the basis of serological tests (23) i.e., Rapid Diagnostic Test NS1 Antigen (AccuDiag™ Dengue NS1 Antigen ELISA) and IgM Antibody detection (Calbiotech Dengue Virus IgM ELISA) as per instructions of the manufacturer. Medical records and demographic data of all 361 Confirmed DF Patients were obtained using a predesigned Performa and analyzed by SPSS (Version-16) (Chicago, IL, USA). Results were expressed in terms of means, standard deviation (SD) and proportions.

## Results

### Epidemiological Findings

**Fig. 1.**
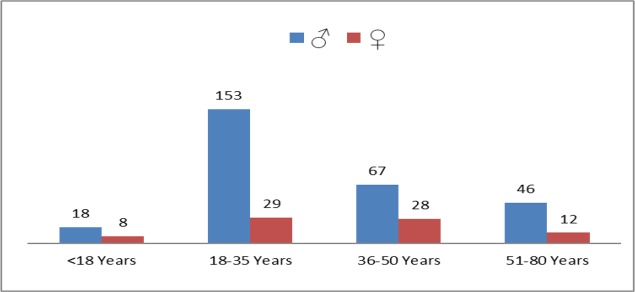
Age and gender ratio of dengue fever patients studied during 1^st^ July to 31^st^ Dec 2015 in Nishtar Hospital Multan, Punjab, Pakistan

Overall, 2477 patients with suspicion of DF were reported at Nishtar Hospital Multan in the year 2015, out of which 1794, 292 and 361 were marked as suspected, probable and confirmed respectively based on serological positivity of IgM and NS1 tests. The current study is based on confirmed endures, comprising 284 (78.67%) males and 77 (21.33%) females. Patients with <18yr of age were 7.2%, 16.1% were >50yr while 26.3% were in the age group of 3–50. The majority of patients were 50.4% and belonged to the age group between 18–35yr (Fig. 1). All the patients had fever from early phase of the illness (mean 5.27d, SD 1.57) while mean hospital stay was 2.64d (SD 1.2). All the patients were recovered and discharged, and no mortality was reported.

### Geographical Distribution

**Fig. 2.**
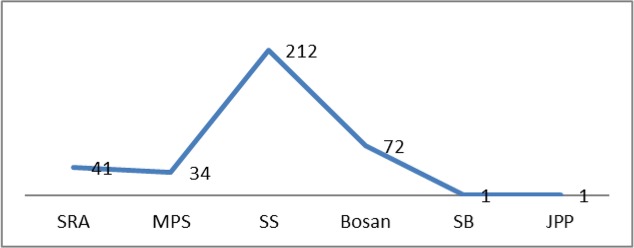
Prevalence of dengue fever patients in different towns of Multan, Pakistan studied during 1^st^ July to 31^st^ Dec 2015

Among the 4 urban and two rural towns of Multan District, the highest number of DF patients was recorded from Sher Shah Town (58.72%), followed by Bosan Town (19.96%), Shah Rukn-e-Alam Town (11.36%) and Musa Pak Shaheed Town (9.42%), while only 0.54% of the patients were reported from the Rural Towns (Fig. 2). From the Confirmed Patients, 99% (355) were residents of Multan District while only 1% (7) was reported from other Districts, whereas 8% (32) had travel history from other districts before onset of fever.

### Clinical findings

**Fig. 3.**
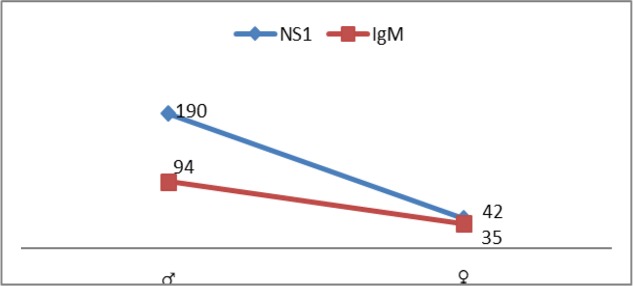
Serological representation of dengue fever patients studied during 1^st^ July to 31^st^ Dec 2015 in Nishtar Hospital Multan, Punjab, Pakistan

All the patients were reported with fever and other symptoms like vomiting, myalgia, headache, arthralgia, epistaxis, loose motion, and abdominal pain. Among all the 361 patients 64.26 (190 men and 42 women) were positive for NS1 antigen, and 35.73% (94 men and 35 women) cases were IgM positive (Fig. 3).

The platelet count in 63 (17.45%) patients was less than 50×10^3^/microliter, in 254 (70.36%) it was between 50×10^3^–100×10^3^/microliter while in 44 (12.18%) patients the count was above 100×10^3^/microliter. White Blood Cells (WBC) count was also recorded on the date of admission in hospital which was ≤ 4×10^3^/microliter in 70.08% (253) and > 4×10^3^/microliter in 29.91% (108) patients.

During the study period the number of patients was highest in the month of October with 287 (79.51%) cases, and a fair reduction was observed in Sep and Nov with 30 (8.31%) and 40 (11.09%) patients, respectively. While only 4 (1.09%) patients were admitted in the months of Jul, Aug and Dec collectively (Table 1).

**Table 1.** Monthly infection rate of dengue fever patients studied during 1^st^ July to 31^st^ Dec 2015 in Nishtar Hospital Multan, Punjab, Pakistan

**Month**	**July**	**August**	**September**	**October**	**November**	**December**
**Patients**	2	1	30	287	40	1

## Discussion

The present study was conducted during the DF outbreak in the year 2015 in Multan District. Infection rate was high in men (78.67%) as compared to women (21.33%).

The similar trends were observed by various workers (22–25), the DF ratio between men and women was 55.3:4.7, 58:42 and 59.3:40.7 (in percentage), respectively. Similar results were observed in Kolkata, India where highest infection rate was reported in males than in females (26). The same tendency was noted in a recent study in Sri Lanka in which 66.2% were males and 33.8% females (27). Males get more infections as compared to females. The main reason for this trend may be that men are more exposed to outdoor activities and environmental factors as compared to females (28). Another reason may be that females wear long sleeves and full trousers in Pakistan which give them protection from mosquito bites (23). It is, therefore, suggested to adopt personal protective measures from mosquito bites.

In the current study, among the age groups, the individuals of 18–35yr of age got maximum infections i.e., 50.41% followed by 26.31% in age group of 36–50, 16% >50yr and 7.2% in <18yr of age. In a similar study, maximum infection rate was observed in the age group of 21–40yr (29), while in another study highest number of patients were reported between 11–30yr (25). The maximum infection rate in age group of 15–45yr was also reported (30). However, contrary to our findings a study showed the highest number of DF patients in the age group of 0–10yr (31). In Puerto Rico, in 1994–1999, elders (≥ 65yr) and infants (1 year) were more infected as compared to the youth (2 to 18yr) and adults (19 to 64yr). Infants and elderly are more delicate and easily carry and develop the infection (32). In the present study, assumption can be made that people between 18–50yr have more outdoor activities and chances of vector contact than the other age groups and thus magnitude of infection is higher in this age group.

In the study under discussion, mean duration of fever was 5.27d (SD 1.57) which was in accordance with the findings of another study i.e., 5.5d, SD 2.7 (33). The mean hospital stay was 2.64d (SD 1.2), which was 4.76±1.53 in another study (28). In a study carried out in Sri Lanka, the same was reported to be 2.7d (SD 0.6) (25). On the contrary, in another work mean hospital stay was reported to be 7–12d (30).

Taking into account the history of infection in the outbreak under discussion, 91% of the patients were local residents with no travel history and only 1% were from other districts, whereas 8% had a travel history to other districts. The maximum infection was acquired locally, which means that the local vector was infected by some means.

The majority of cases were reported in the month of Oct i.e., 79.51%, with 8.31% in Sep and 11.09% in Nov, while only 1.09% of positive DF cases were reported in the rest of the 3 months (July, Aug and Dec). In District Swat, KPK of Pakistan the same pattern was observed. The rate of infection was low in the month of July and with gradual increase. The highest number was reported in Oct i.e., 36.11 %. The increasing number of patients was associated with the pattern of rain (29). Dengue outbreaks are clearly linked with rainy season (34). Patient count was zero from Jan to Mar, with only 8 reported from Apr to Aug. The patient count started to rise, and the maximum number of confirmed DF cases were reported from Sept to Nov and again declined in Dec. In the same description, maximum numbers of patients were observed in the months from Aug to Oct 2006 which was a period of heavy rainfall (20).

All these findings are in accordance with the current study and the disease incidence is clearly related to the monsoon and pre-monsoon period. The high rainfall provides increased number of potential breeding sites for vector mosquitoes, consequently increase in number of DF cases. The number of patients was also linked to increased temperature and rainfall, and during high-temperature mosquito got the chances to feed more on humans as compared to low-temperature season (23). Mosquito population is linked with raised temperatures (35) and increase in temperature by 1 °C resulted in increase the risk of DF transmission by 1.95 times (36).

Among the four urban and two rural towns of Multan District, the highest number of patients (212) was reported from Sher Shah Town. The reason for this might be the maximum number of tyre shops present in the town. Tyres serve as breeding place for mosquitoes and the same can serve as a mean of shipping the dengue vectors in and outside an area (23). Moreover, Multan is situated almost in the center of the country and serves as main perching place on major roads and rail track. The city is connected by rail with all parts of the country and lies on the main track between Karachi, Peshawar, Lahore and Quetta. For the whole year, people travel through the city from all around the state. In year 2015, Karachi, Lahore and Rawalpindi were also under dengue outbreak. The presence of Railway Station in Sher Shah town can be a potential reason for more dengue burden in the locality under discussion. The evidence supporting the statement is that a large number of DF patients were the residents of Railway Colony Multan located around the Railway Station of Sher Shah town. The movement of infected persons is one of the main causes of the swift spread of this disease in Pakistan (20).

Taking into account the clinical aspects, all the patients were reported with high-grade fever which is the clear sign of the disease. These observations are in accordance with the studies carried out by different workers (25–29). In the outbreak under consideration, not a single patient was diagnosed with DHF or DSS, all endure were fully recovered and discharged from the hospital. The reason for this may be easy and in time access of the infected patients to the medical facilities and adaptation of proper remedial measures. All this was assured by the Epidemic Prevention and Control Program in the District.

The blood samples of the patients were confirmed through IgM and NS1 positivity. NS1 Antigen test is used for early detection of infection. This Antigen is detected from 1^st^ to 5^th^ day from onset of fever. Whereas IgM is an antibody produced in mammals in response to any infection and it takes at least one week to formulate. It can be detected from 5^th^ to 9^th^ day from onset of fever (37). In the present study among the confirmed patients, 64.26% reflected the positivity for NS1 while the rest were positive for IgM antibodies. In a study conducted in 2013 with 62 patients, 38 cases were positive for NS1 and 24 were negative. On the other hand, out of 24 NS1 negative tests, 6 patients revealed the positivity of IgM and the rest 18 were found to be negative for IgM antibodies (26). Similarly, it was scrutinized in an experiment that out of 6000 confirmed DF cases 4121 were positive for NS1 (28). In a recent study carried out in India, 23% were NS1 positive (38).

Platelet count was also taken into consideration in the present study. In 17.45% cases platelet count was < 50× 10^3^/microliter, while it was between 50× 10^3^–100× 10^3^/microliter in maximum patients i.e., 70.36%, while in rest of the patients it was > 100× 10^3^/microliter. The same was found to be < 50× 10^3^/microliter in 15.16%, between 50× 10^3^–100× 10^3^/microliter in 28.65% and > 100× 10^3^/microliter in 56.18 % (29). While others reported the platelet count <100× 10^3^/microliter in 55%, 82% and 89% patients respectively (29, 39, 40).

## Conclusion

Epidemiology and the clinical manifestations of the DF patients were revealed and observed that the disease is most prevalent in adult males. The reason behind this is more outdoor activities of adult males and hence more exposure to the disease vector. Therefore, vector control measures should be especially adopted at workplaces. On the clinical side, all the cases were of DF, with no report of DHF or DSS which is a clear indication of timely reporting and better management practices. Same pattern is suggested to be followed to avoid mortalities and reduce the risk factors of the disease.

There is no multivariate vaccine available for control of the disease and the only way to avoid the infection is preventive measures. In this context, community participation and awareness regarding better preventive and control measures of dengue vector is necessary. There is also need for large scale entomological and epidemiological surveys. Otherwise, DF is likely to become a much serious health issue in Pakistan.
